# Outcome study of the Pipeline Vantage Embolization Device (second version) in unruptured (and ruptured) aneurysms (PEDVU(R) study)

**DOI:** 10.1136/jnis-2023-020754

**Published:** 2023-12-09

**Authors:** Thomas C Booth, Ahmed Bassiouny, Jeremy Lynch, Hemant Sonwalkar, Aaron Bleakley, Ahmed Iqbal, Thais Minett, Jonathon Buwanabala, Ana Paula Narata, Tufail Patankar, Fathallah Ismail Islim, Naga Kandasamy, Parthiban Balasundaram, Sara Sciacca, Juveria Siddiqui, Daniel Walsh, Christos Tolias, Ahilan Kailaya-Vasan, Amina A Sultan, Mahmoud Abd El-Latif, Alex Mortimer, Anand Sastry

**Affiliations:** 1 School of Biomedical Engineering & Imaging Sciences, King's College, London, UK; 2 Department of Neuroradiology, King's College Hospital NHS Foundation Trust, London, UK; 3 Diagnostic Radiology, Faculty of Medicine, Mansoura University, Mansoura, Egypt; 4 Department of Interventional Neuroradiology, Royal Preston Hospital, Preston, UK; 5 Department of Neuroradiology, Royal Preston Hospital, Lancashire, UK; 6 Queen Elizabeth University Hospital, Glasgow, UK; 7 Cambridge University Hospitals NHS Foundation Trust, Cambridge, UK; 8 Department of Neuroradiology, University Hospital of Southampton, Southampton, UK; 9 Interventional Neuroradiology, Leeds Teaching Hospitals NHS Trust, Leeds, UK; 10 Department of Interventional Radiology, Leeds General Infirmary, Leeds, UK; 11 Department of Neurosurgery, King's College Hospital NHS Foundation Trust, London, UK; 12 Neuroradiology, North Bristol NHS Trust, Bristol, UK; 13 Radiology Department, University Hospital of Wales, Cardiff and Vale University Health Board, Cardiff, UK

**Keywords:** Aneurysm, Flow Diverter, Hemorrhage, Subarachnoid, Intervention

## Abstract

**ABSTRACT:**

**Background:**

The Pipeline Vantage Embolization Device (PEDV) is the fourth-generation pipeline flow diverter for intracranial aneurysm treatment. There are no outcome studies for the second PEDV version. We aimed to evaluate safety and efficacy outcomes. Primary and secondary objectives were to determine outcomes for unruptured and ruptured cohorts, respectively.

**Methods:**

In this multicenter retrospective and prospective study, we analyzed outcome data from eight centers using core laboratory assessments. We determined 30-day and ≥3-month mortality and morbidity rates, and 6- and 18-month radiographic aneurysm occlusion rates for procedures performed during the period July 2021–March 2023.

**Results:**

We included 121 consecutive patients with 131 aneurysms. The adequate occlusion rate for the unruptured cohort at short-term and medium-term follow up, and also for the ruptured cohort at short-term follow up, was >90%. Two aneurysms (1.5%) underwent retreatment. When mortality attributed to a palliative case in the unruptured cohort, or subarachnoid hemorrhage in the ruptured cohort, was excluded then the overall major adverse event rate in respective cohorts was 7.5% and 23.5%, with 0% mortality rates for each. When all event causes were included on an intention-to-treat basis, the major adverse event rates in respective cohorts were 8.3% and 40.9%, with 0.9% and 22.7% mortality rates.

**Conclusions:**

For unruptured aneurysm treatment, the second PEDV version appears to have a superior efficacy and similar safety profile to previous-generation PEDs. These are acceptable outcomes in this pragmatic and non-industry-sponsored study. Analysis of ruptured aneurysm outcomes is limited by cohort size. Further prospective studies, particularly for ruptured aneurysms, are needed.

**What is already known on this topic**

- There are no published outcome studies for the globally-released second version of the Pipeline Vantage Embolization Device (PEDV) which has superseded the first version that was released to only a few testing centers.

**What this study adds**

- For unruptured aneurysm treatment, the second PEDV version appears to have a superior efficacy (radiological occlusion rate) and similar safety profile to previous-generation Pipeline Embolization Devices. For the treatment of ruptured aneurysms, conclusions are limited due to the cohort size; however, observations are that efficacy appears similar to previous studies and may be acceptable, but that there appears to be a high rate of adverse events.

**How this study might affect research, practice or policy**

- Overall, these are acceptable outcomes for unruptured aneurysm treatment in this pragmatic and non-industry-sponsored study. To justify the routine use of PEDVs for ruptured aneurysms, evidence from further prospective outcome studies is needed.

## Introduction

 For more than a decade, there has been a steady increase in the indications for use of flow diverter devices in the treatment of aneurysms.^1^ The first-generation Pipeline Embolization Devices (PEDs) were used increasingly following their introduction in 2011 with proven safety and efficacy (radiological occlusion rates).^2 3^ The second-generation (Pipeline Flex Embolization Device) improved the ability to reposition and redeploy the PED,^4^ while the third-generation (PED with Shield Technology) was coated with phosphorylcholine in order to reduce stent thrombogenicity and had similar efficacy (occlusion rates) and safety outcomes compared with previous generations.^5 6^ Further refinements resulted in the latest (fourth) generation (Pipeline Vantage Embolization Device (PEDV)) and aimed to improve delivery, distal opening, wall apposition, and ease of re-sheathing.^7 8^ The first limited-release version of PEDV was discontinued following deployment difficulties^7^ and replaced by a second version which is now the only commercially available PED (online supplementary fig. S1). Our study aim was to assess short-term and medium-term safety and efficacy (occlusion rates) outcomes of the second version. The primary and secondary objectives were to determine these outcomes for unruptured and ruptured cohorts, respectively.

## Methods

A multicenter, pragmatic study was performed in eight UK centers from July 2021 to March 2023. The inclusion criteria were all patients ≥18 years old who underwent PEDV aneurysm treatment. Centers used local protocols for patient selection, treatment, and follow up. We captured consecutive prospective clinical and radiological follow-up data. We also captured consecutive retrospective treatment data with details including multidisciplinary team make-up, population at risk, procedural details, and anticoagulation and antiplatelet protocols. The study was performed in accordance with the 1964 Declaration of Helsinki and later amendments (UK Research Ethics Committee and Health Research Authority IRAS 317314).

### Study endpoints

The primary safety endpoint was the cumulative occurrence of major adverse events at all follow-up timepoints. The primary efficacy endpoint was adequate occlusion at short-term, 6-month (range 3–9 months) follow up. The secondary efficacy endpoint was adequate occlusion at medium-term, 18-month (12-30) follow up. Further definitions are provided below.

### Patient details

Data collected included age at presentation and gender. In the ruptured aneurysm cohort, the Hunt and Hess scale was used to assess the pre-operative clinical state of the patient.

### Aneurysm details

We collected detailed data on rupture status, previous treatment, location, size, and morphology. Data included aneurysm type (true or pseudoaneurysm), shape (saccular, fusiform, or blister), smooth or irregular (lobulated/daughter sac), whether located in sidewall or bifurcation, and whether thrombosed. Aneurysm measurements included neck, dome width, height, and maximum diameter. Dome-to-neck, width-to-height, and aspect ratios were calculated for all aneurysms. Details were similarly collected for any secondary (non-target) aneurysms treated by the same PEDV during the same session.

### Procedure details

We recorded access routes (radial or femoral), catheters used, data on device deployment failure, and adjuncts used (eg, coiling or balloon angioplasty). O’Kelly Marotta scaling (OKM) was determined immediately after PEDV deployment.^9^

### Adverse events

Adverse events were classified according to International Retrospective Study of the Pipeline Embolization Device (IntrePED) methodology.^10^ “Neurologic adverse events” included subarachnoid hemorrhage (SAH), intraparenchymal hemorrhage, ischemic stroke, parent artery stenosis, and cranial neuropathy. Complications were considered “peri-procedural” if occurring <30 days after embolization and “post-procedural” if occurring ≥30 days. A persistent clinical deficit at 7 days following the event was defined as a “major” adverse event. Other events that resolved within 7 days with no clinical sequelae were defined as “minor” adverse events.

### Follow-up imaging

Digital subtraction angiography (DSA), computed tomography angiography (CTA), and magnetic resonance angiography (MRA) were used alone or in combination according to local center protocol. Follow-up imaging was performed at short- and medium-term. A Modified Raymond Roy Classification (MRRC) scale of I or II was considered “adequate occlusion”; and IIIa or IIIb “inadequate occlusion” (online supplementary fig. S2).^11^ OKM was used additionally in cases with follow-up DSA. In-stent stenosis and patency of aneurysmal arterial branches were assessed. In-stent stenosis was graded as 0%, <50% stenosis, or ≥50% stenosis. Cross-sectional imaging allowed longitudinal axial aneurysmal size comparisons,^12^ and interval increases were recorded with a threshold of >2 mm. Retreatment rates were determined.

### Statistical analysis

Both core laboratory and local reader outcome assessments were made for each case. Inter-rater reliability was determined using weighted Cohen’s kappa.^13^ For all discrepant readings, core laboratory assessments were used. Outcomes were calculated on both a complete per-protocol basis (device deployed and followed up) and an intention-to-treat (ITT) basis (included device not deployed or patient deceased before follow up). Aneurysm characteristics and occlusion rates were performed on a per-aneurysm basis because some patients had >1 aneurysm treated with ≥1 PEDV. Fisher exact tests (and the Freeman–Halton extension) were employed for comparative statistics. Descriptive and comparative statistics were calculated using SPSS (IBM Version 26.0).

## Results

Eight centers were included in the study (six in England, one in Scotland, and one in Wales) with a mean population-at-risk of 2.25 million per center catchment area. All cases were selected following consensus at local neurovascular multidisciplinary team meetings which included senior (UK consultant grade; US attending equivalent) interventional neuroradiologists (median three per center) and neurovascular surgeons (median two per center). The operators had 9.1±5.9 years (mean±SD; range 2–25 years) of experience.

### Patients and aneurysm characteristics

The unruptured cohort included 108 patients with 116 aneurysms on an ITT basis. The PEDV could not be deployed safely in 3/108 (2.8%) patients with 4/116 (3.4%) aneurysms. One terminally-ill patient 1/108 (0.9%) palliated for a partially thrombosed fusiform basilar artery giant aneurysm died within the peri-procedural time window before follow-up imaging, giving 104 patients with 111 aneurysms for complete per-protocol analysis (table 1). The ruptured cohort included 22 patients with 25 aneurysms on an ITT basis, with 17 patients with 20 aneurysms available for complete per-protocol analysis. In this cohort, 5 patients with five aneurysms died from SAH complications within the peri-procedural time window before follow-up imaging. Of all the patients treated with PEDV, 37/121 (30.6%) were retreatments (36 previous endovascular procedures (coiling, intrasaccular device implantation, stenting), 1 open surgery (wrapping)). Single aneurysms were treated in 111/121 (91.7%) procedures, and two aneurysms were treated in 10/121 (8.3%) procedures.

**Table 1:** Patient and aneurysm characteristics (per-protocol analysis)

Characteristic	Unruptured cohort		Ruptured cohort	
Patient age	57.3±13.9 years (21–86 years)		55.9±14.5 years (29–83 years)	
Gender	Males	Females	Males	Females
	29 (27.9%)	75 (72.1%)	5 (29.4%)	12 (70.6%)
Number of aneurysms treated by the same PEDV	Single	Multiple	Single	Multiple
	97 (93.3%)	7 (6.7%)	14 (82.4%)	3 (17.6%)
Aneurysm location*	ICA para-ophthalmic: 36 (32.4%)		ICA para-ophthalmic: 4 (20.0%)	
	ICA PCOM: 23 (20.7%)		MCA: 3 (15.0%)	
	ICA cavernous: 16 (14.4%)		PICA: 3 (15.0%)	
	Ophthalmic: 14 (12.6%)		ICA PCOM: 2 (10.0%)	
	MCA: 6 (5.4%)		Ophthalmic: 2 (10.0%)	
	ACOM: 5 (4.5%)		ICA cavernous: 1 (5.0%)	
	Basilar: 3 (2.7%)		ICA choroidal: 1 (5.0%)	
	PICA: 2 (1.8%)		ICA terminal: 1 (5.0%)	
	ICA choroidal: 2 (1.8%)		ACA: 1 (5.0%)	
	ICA terminal: 1 (0.9%)		ACOM: 1 (5.0%)	
	ACA: 1 (0.9%)		PCA: 1 (5.0%)	
	ICA cervical: 1 (0.9%)			
	Vertebral: 1 (0.9%)			
Anterior or posterior circulation	Anterior: 105 (94.6%)	Posterior: 6 (5.4%)	Anterior: 16 (80.0%)	Posterior: 4 (20.0%)
Sidewall/bifurcation	Sidewall: 96 (86.5%)	Bifurcation: 15 (13.5%)	Sidewall: 15 (75.0%)	Bifurcation: 5 (25.0%)
Arterial branch originating from aneurysm sac	27 (24.3%)		5 (25.0%)	
Hunt and Hess scale	N/A		1: 7 (41.2%)	
			1 a: 1 (5.9%)	
			2: 3 (17.6%)	
			3: 6 (35.3%)	
Thrombosed aneurysms	Partially thrombosed: 17 (15.3%)		None (0%)	
Shape	Saccular: 103 (92.8%)		Saccular: 16 (80.0%)	
	Fusiform: 6 (5.4%)		Fusiform: 1 (5.0%)	
	Blister: 2 (1.8%)		Blister: 3 (15.0%)	
Aneurysm type	True aneurysm: 107 (96.4%)		True aneurysm: 18 (90%)	
	Pseudo-aneurysm: 4 (3.6%)		Pseudo-aneurysm: 2 (10%)	
Aneurysms with lobular morphology or daughter sac	37 (33.3%)		6 (30%)	
Aneurysm measurements (mm or ratio)				
Neck	4.8+2.1 (1.2–12)		3.1+1.7 (1.0–7.2)	
Dome width	8.2+6.3 (1–37)		4.4+5.0 (1.5–23.3)	
Height	8.4+6.2 (1–35.5)		3.6+3.6 (1.0–16.2)	
Maximum diameter	9.6+6.7 (1.5–37)		4.9+4.8 (1.5–23.0)	
Mean dome-to-neck (W/N)	1.7+1.7 (0.7–14.0)		1.3+0.6 (0.7–3.2)	
Mean aspect ratio (H/N)	1.7+1.1 (0.4–6.3)		1.2+0.6 (0.3–2.5)	
Mean width-to-height (W/H)	1.0+0.4 (0.4–2.8)		1.3+0.6 (0.6–3.0)	
Aneurysm class†				
Small aneurysms	26 (23.4%)		12 (60.0%)	
Medium aneurysms	44 (39.6%)		6 (30.0%)	
Large aneurysms	37 (33.3%)		2 (10.0%)	
Giant aneurysms	4 (3.6%)		0 (0.0%)	
Wide-neck aneurysms	60 (54.1%)		4 (20.0%)	

Data are presented aindicated as mean±standard deviation, range or absolute number of cases (relative frequency in %).

Relative frequency for patient gender and number of aneurysms treated by the same PEDV is related to the number of patients (n=104 for unruptured and n=17 for ruptured).

Relative frequency for the remaining data is related to the number of aneurysms (n=111 for unruptured and n=20 for ruptured).

* ACA, anterior cerebral artery (distal to the ACOM); ACOM, anterior communicating artery; ICA, internal carotid artery; MCA, middle cerebral artery; PCA, posterior cerebral artery; PICA, posterior inferior cerebellar artery. NYU ICA anatomical classification used.^21^

† Aneurysms were classified into small (<5 mm), medium, large (≥10 mm), and giant aneurysms (≥25 mm). Wide- neck aneurysms defined as neck >4 mm.^22^

N/Anot applicablePEDVPipeline Vantage Embolization Device

### Antiplatelets and anticoagulation

Regimens varied between center pre- and post-treatment (online supplemental table S1). Four centers (50%) routinely performed platelet resistance testing using VerifyNow (Accriva Diagnostics, San Diego, CA, USA). One center (12.5%) performed testing only when clopidogrel was used.

### Treatment

Interventional approaches and catheter details are summarized in online supplemental table S2. The most common guide catheter for a transfemoral approach was the Neuron MAX (Penumbra, Alameda, CA, USA) and for a transradial approach the Benchmark (Penumbra). Implanted PEDV sizes ranged from 2.5×10 to 6.0×50 mm. Adjunctive coiling was performed in 41/121 (33.9%) patients. In-stent balloon angioplasty was performed in 20/121 (16.6%) patients. Two patients (1.7%) underwent adjunctive Woven EndoBridge (WEB) (MicroVention, Aliso Viejo, CA, USA) implantation. Immediate postinterventional occlusion rates are shown in online supplemental table S3.

### Clinical outcomes

Six patients (6/130, 4.6%) died in the peri-procedural period on an ITT basis (5/6, 83.3% from presenting SAH). All surviving patients (124/124) had a minimum of ≥3 months clinical follow up of which 88/124 (71.0%) had ≥6 months follow up (online supplemental table S4).

#### Safety endpoints

The primary safety endpoint (cumulative major adverse event rate) was 8.3% (9/108) patients with unruptured aneurysms on an ITT basis (table 2). The mortality rate was 0.9% (1/108) on an ITT basis. All major adverse events were neurological. There was one major adverse event ≥3 months and no adverse events ≥6 months. On a per-protocol basis, the cumulative major adverse event rate was 7.7% (8/104) and the mortality rate was 0% (detailed below); if the three patients requiring alternative treatment for failed PEDV deployment are added, the respective rates were 7.5% (8/107) and 0%.

**Table 2:** Clinical outcomes (intention-to-treat analysis unless otherwise described)

Outcome	Unruptured cohort (n=108)	Major or minor
**Periprocedural**		
Adverse events (no neurological sequelae)	Difficulty deploying PEDV: 6 (5.6%)Groin hematoma: 4 (3.7%)Failure to deploy PEDV*: 3 (2.8%)Transient thrombus: 2 (1.9%)Extracranial ICA dissection: 1 (0.9%)	16 Minor
Neurological adverse events	Ischemic stroke: 7 (6.5%)Cranial neuropathy: 2 (1.9%)Intraparenchymal hemorrhage: 1 (0.9%)Death†: 1 (0.9%)	2 Major & 5 Minor2 Major1 Major1 Major
Total complications (ITT analysis)		Minor: 21 (19.6%)Major: 6 (5.6%)
Total complications (per-protocol basis‡)		Minor: 21 (20.2%)Major: 5 (4.8%)
Total complications (per-protocol basis and additionally including those with failed PEDV deployment§)		Minor: 21 (19.6%)Major: 5 (4.7%)
**Postprocedural**		
Adverse events (no neurological sequelae)	N/A	
Neurological adverse events	Ischemic stroke: 3 (2.8%)Cranial neuropathy: 1 (0.9%)Parent artery occlusion¶: 1 (0.9%)Aneurysm growth with mass effect: 1 (0.9%)	3 Minor1 Major1 Major1 Major
Total complications (ITT analysis)		Minor: 3 (2.8%)Major: 3 (2.8%)
Total complications (per-protocol basis‡)		Minor: 3 (2.9%)Major: 3 (2.9%)
Total complications (per-protocol basis and additionally including those with failed PEDV deployment§)		Minor: 3 (2.8%)Major: 3 (2.8%)
	**Ruptured cohort (n=22**)	
Peri-procedural		
Adverse events (no neurological sequelae)	Groin hematoma: 1 (4.5%)Extracranial ICA dissection: 1 (4.5%)Difficulty deploying PEDV: 1 (4.5%)	3 Minor
Neurological adverse events	Death**: 5 (22.7%)Ischemic stroke**: 4 (18.2%)Second subarachnoid hemorrhage††: 1 (4.5%)	5 Major3 Major & 1 Minor1 Major
Total complications (ITT analysis)		Minor: 4 (18.2%)Major: 9 (40.9%)
Total complications (per-protocol basis‡‡)		Minor: 4 (23.5%)Major: 4 (23.5%)
Post-procedural		
Adverse events (no neurological sequelae)	N/A	
Neurological adverse events	N/A	

Peri-procedural, occurring <30 days after embolization. Post-procedural, occurring ≥30 days after embolization.

"Major” adverse event: a persistent clinical deficit at 7 days following the event. “Minor” adverse event: events that resolved within 7 days with no clinical sequelae.

* Unclear why two2 PEDVs failed to deploy, but appeared to be entirely technical. There was in-stent stenosis from a previous stenting which may have contributed to failure to deploy PEDV in the third case. For the 2two purely technical failures, both cases were cancelled with 1one patient undergoing subsequent parent vessel occlusion and 1one patient undergoing subsequent p64 (Phenox, Bochum, Germany) flow diversion. In the third case where there was in-stent stenosis, the patient underwent coiling during the same session.

† Adjudication concluded death inevitable without PEDV implantation.

‡ Denominator n=104.

§ Denominator n=107. Includes three patients patients requiring alternative treatment.

¶ No neurological clinical features but included as neurological adverse event according to IntrePED methodology.^10^

** Adjudication concluded death more likely to be attributable to presenting subarachnoid hemorrhage than PEDV implantation; data wereas less clear for ischemic stroke.

†† Patient had ruptured ICA para-ophthalmic aneurysm. Pre--operative intravenous tirofiban with infusion. Underwent PEDV deployment, adjunctive coiling and balloon angioplasty. Loaded with aspirin and clopidogrel. Re-rupture with immediate hydrocephalus in recovery. Given two2 pools of platelets and underwent extra ventricular drain insertion. Subsequently underwent ventriculo-peritoneal shunt insertion. Complete occlusion of aneurysm at follow up.

‡‡ Denominator n=17.

ICAinternal carotid arteryITTintention-to-treat N/Anot applicablePEDVPipeline Vantage Embolization Device

Clinical outcomes for ruptured aneurysms were assessed as a secondary objective (table 2). The primary safety endpoint (cumulative major adverse event rate) was 9/22 (40.9%) patients on an ITT basis. The mortality rate was 5/22 (22.7%) on an ITT basis. All major adverse events were neurological, and there were no adverse events ≥30 days. On a per-protocol basis, the cumulative major adverse event rate was 23.5% (4/17) and mortality rate was 0% (detailed below).

### Peri-procedural adverse events (without neurological sequelae)

PEDV deployment failure occurred only within the unruptured patient cohort (3/108, 2.8%) procedures (detailed in table 2, including subsequent endovascular treatments). Operators reported that PEDV deployment was challenging in 6/108 (5.6%) and 1/22 (4.5%) of unruptured and ruptured procedures, respectively, due to PEDV proximal migration, shortening, herniation into aneurysm, or improper sizing, or due to inadequate large catheter support. In all seven cases, the PEDV was deployed without clinical complication. Other adverse events without neurological sequelae were intraprocedural thrombi formation which were treated therapeutically, extracranial ICA dissections which did not appear to be stenotic and were treated therapeutically, and groin hematomas which were managed conservatively.

### Peri-procedural neurological adverse events in the unruptured cohort

Seven patients (7/108, 6.5%) had ischemic strokes, two of which lasted more than 7 days and were categorized as major complications. Two patients (2/108, 1.9%) had cranial neuropathies. One in the form of horizontal diplopia which started on day 17 after the procedure and lasted for 10 days. The other had a right retinal branch artery occlusion presenting on day 7 after the procedure with right eye pain and persistent visual loss. Another patient had hemorrhagic transformation of an asymptomatic subcortical ischemic infarct on day 24 after the procedure. Clinical features resolved after 8 days and the patient was discharged. A patient who presented with worsening cognition, gait imbalance, and confusion due to a thrombosed fusiform basilar artery giant aneurysm underwent palliative stepwise Leo stent (Balt, Montmorency, France) placement followed by a subsequent PEDV and coiling procedure. The patient developed a fatal SAH 3 days after the final procedure. While adjudication concluded that imminent death was inevitable regardless of PEDV implantation in this terminally-ill patient,^14 15^ arguably the ITT-derived mortality rate better represents real-world use.

### Post-procedural neurological adverse events in the unruptured cohort

Three patients (3/108, 2.8%) had ischemic strokes that resolved within 7 days. One patient had a cranial neuropathy on day 55 after the procedure presenting with headache and left temporal hemianopia which resolved with steroids after 14 days. One patient who underwent stent-within-stent deployment developed delayed asymptomatic occlusion of the left intracranial ICA diagnosed on imaging on day 64 after the procedure. One patient developed clinical features suggestive of meningitis 165 days after the procedure; however, cross-sectional imaging showed 10 mm enlargement of the thrombosed component of the aneurysm and mass effect. This was successfully managed with a reduction from dual to single antiplatelet therapy with a corresponding cessation of aneurysm growth and resolution of the surrounding edema.

### Periprocedural neurological adverse events in the ruptured cohort

All complications in this cohort were peri-procedural. Five patients had a poor initial presentation (Hunt and Hess 4–5) and died shortly after the procedure. These cases were included on an ITT basis but excluded from per-protocol analyses as adjudication concluded that death was likely to be attributable to the presenting SAH without contribution from PEDV implantation. Four patients (4/23, 17.4%) developed ischemic stroke, three of which were categorized as major complications. Adjudication was inconclusive for the cause of the ischemic strokes and the data were not excluded from per-protocol analyses. One patient re-ruptured, developed hydrocephalus, and underwent extraventricular drain insertion (procedure and complication management detailed in table 2).

For all neurological events, unruptured and ruptured, we have shown the association between which antiplatelet therapy was used in these patients, and whether VerifyNow was employed (online supplemental table S5).

### Follow-up imaging and aneurysm occlusion

There was variation in imaging follow-up protocols between centers (online supplemental table S6). DSA was used preferentially in some centers if the aneurysm had been coiled, or if in-stent stenosis was suspected but indeterminate on MRA due to susceptibility artefact. To determine the suitability of MRA for PEDV follow-up imaging, we also compared those follow-up DSAs (considered reference standard) and MRAs (considered index test) that were performed within 3 months of one another. Nine aneurysms from six centers (mean imaging interval 2.1 months) were included with perfect agreement (kappa=1.0) in MRRC assessments (time-of-flight and contrast-enhanced MRA were used eight times and once, respectively). Therefore, MRA was considered suitable for the estimation of accurate occlusion rates alongside DSA. When MRA and DSA were compared for in-stent stenosis assessment, there was a 6/9 (66.6%) discrepancy rate as MRA was considered uninterpretable due to susceptibility (online supplemental fig. S3); 2/9 (22.2%) patients demonstrated <50% stenosis on DSA but were uninterpretable on MRA. Therefore, MRA was not considered accurate for estimating in-stent stenosis.

### Efficacy endpoints

#### Aneurysm occlusion

Short-term follow-up imaging (6 months) was available for 110/111 unruptured aneurysms. The primary efficacy endpoint was 100/110 (90.9%) aneurysms adequately occluded on a per-protocol basis (table 3). On an ITT basis, we classified the one aneurysm without short-term follow-up imaging as inadequately occluded, and we included the four aneurysms which failed flow diversion (all of which had available short-term follow-up imaging) to give 104/115 (90.4%) aneurysms that met the efficacy endpoint. The complete occlusion rate (MRRC 1) was 70.9% (78/110) and 71.3% (82/115) on a per-protocol and ITT basis, respectively.

**Table 3:** Unruptured aneurysms imaging follow up (per-protocol analysis).

DSA at 6 months (range 3–9 months)	I		II		IIIa		IIIb				Total	Interobserver assessment*
												(Kappa score†)
Modified Raymond Roy DSA	7		4		1		2				14	0.94 (almost perfect agreement)
	I		II		III							
Raymond Roy DSA	7		4		3						14	0.92 (almost perfect agreement)
	Yes				No							
Adequate occlusion or not	11				3						14	0.81 (almost perfect agreement)
	A1	A2	A3	B1	B2	B3	C1	C2	C3	D		
OKM	0	0	0	0	1	2	2	1	1	7	14	0.81 (almost perfect agreement)

MRA at 6 months (range 3–9 months)	I	II	IIIa	IIIb	Total	
Modified Raymond Roy MRA	69	17§	6	2	94	0.90 (almost perfect agreement)
	I	II	III			
Raymond Roy MRA	69	17§	8		94	0.89 (almost perfect agreement)
	Yes		No			
Adequate occlusion or not	86		8		94	0.83 (almost perfect agreement)

CTA at 6 months (range 3–9 months)	I	II	IIIa	IIIb	Total	
Modified Raymond Roy CTA	4	5	0	0	9	0.69 (substantial agreement)
	I	II	III			
Raymond Roy CTA	4	5	0		9	0.79 (substantial agreement)
	Yes		No			
Adequate occlusion or not	9		0		9	0.0 (no agreement)‡

All imaging at 6 months (range 3–9 months)	I	II	IIIa	IIIb	Total	
Modified Raymond Roy	78	22	6	4	110	N/A
	I	II	III			
Raymond Roy	78	22	10		110	N/A
	Yes		No			
Adequate occlusion or not	100		10		110	N/A

DSA at 18 months (range 12–30 months)	I		II		IIIa		IIIb				Total	
Modified Raymond Roy DSA	7		1§		0		0				8	1.0 (perfect agreement)
	I		II		III							
Raymond Roy DSA	7		1§		0						8	1.0 (perfect agreement)
	Yes				No							
Adequate occlusion or not	8				0						8	1.0 (perfect agreement)
	A1	A2	A3	B1§	B2	B3	C1	C2	C3	D		
OKM	0	0	0	1	0	0	0	0	0	7	8	1.0 (perfect agreement)

MRA at 18 months (range 12–30 months)	I	II	IIIa	IIIb	Total	
Modified Raymond Roy MRA	3	1	1	0	5	0.74 (substantial agreement)
	I	II	III			
Raymond Roy MRA	3	1	1		5	0.74 (substantial agreement)
	Yes		No			
Adequate occlusion or not	4		1		5	1.0 (perfect agreement)

All imaging at 18 months (range 12–30 months)	I	II	IIIa	IIIb	Total	
Modified Raymond Roy	10	2	1	0	13	N/A
	I	II	III			
Raymond Roy	10	2	1		13	N/A
	Yes		No			
Adequate occlusion or not	12		1		13	N/A

* Two readings, one1 core laboratory and other by a local reader, were compared. For all discrepant readings, the core laboratory assessment was used. The data shows that estimates of occlusion rates were accurate when performed locally for MRA and DSA.

† Inter-reader reliability used weighted (or unweighted for 2×2) Cohen’s kKappa. N/A: nNot applicable for ‘All iImaging’ as data contains some DSA and MRA cases for same patient.

‡ On account of two2 discrepancies of IIIa (local reader) and II (core laboratory), statistically a chance finding. Clearly, more granular scores are statistically more informative.

§ Includes one PEDV re-treatment patient. Patient had a previously-ruptured ICA PCOM aneurysm which was treated with coiling. Due to MRRC II remnant, patient subsequently re-treated with PEDV (and included in current study). After PEDV deployment (with some foreshortening included as an adverse event in table 2), short-term imaging follow up with MRA showed MRRC II remnant which persisted at longmedium-term imaging follow up with DSA. Patient underwent a second re-treatment whichwith a Flow-Redirection Endoluminal Device (FRED; MicroVention, Aliso Viejo, CA, USA).

CTA, computed tomography angiography; DSA, digital subtraction angiography; ICAinternal carotid arteryMRA, magnetic resonance angiography; MRRCModified Raymond Roy ClassificationN/Anot applicableOKM, O’Kelly Marotta gradingPCOMposterior communicating artery PEDVPipeline Vantage Embolization Device

Medium-term follow-up imaging (18 months) was available for 13/111 unruptured aneurysms (including the one aneurysm without short-term follow-up imaging). The secondary efficacy endpoint was 12/13 (92.3%) aneurysms adequately occluded on a per-protocol basis (table 3). The complete occlusion rate (MRRC 1) was 10/13 (76.9%) on a per-protocol basis (no additional ITT cases). Of the 12/13 aneurysms that could be compared longitudinally between the two follow-up timepoints, 10/12 (83.3%) aneurysms were stable and 2/12 (16.7%) improved from MRRC 2 to MRRC 1.

Short-term follow-up imaging for 20/20 ruptured aneurysms was assessed as a secondary objective. The primary efficacy endpoint was 18/20 (90.0%) aneurysms adequately occluded on a per-protocol basis (table 4). The complete occlusion rate (MRRC 1) was 80% (16/20). There was no medium-term follow-up imaging.

**Table 4:** Ruptured aneurysm imaging follow up (per-protocol analysis)

DSA at 6 months (range 3–9 months)	I		II		IIIa		IIIb				Total	Interobserver assessment*
												(Kappa score†)
Modified Raymond Roy DSA	5		1		0		0				6	1.0 (perfect agreement)
	I		II		III							
Raymond Roy DSA	5		1		0						6	1.0 (perfect agreement)
	Yes				No							
Adequate occlusion or not	6				0						6	1.0 (perfect agreement)
	A1	A2	A3	B1	B2	B3	C1	C2	C3	D		
OKM	0	0	0	0	1	0	0	0	0	5	6	1.0 (perfect agreement)

MRA at 6 months (range 3–9 months)	I	II	IIIa	IIIb	Total	
Modified Raymond Roy MRA	14	1	2	0	17	1.0 (perfect agreement)
	I	II	III			
Raymond Roy MRA	14	1	2		17	1.0 (perfect agreement)
	Yes		No			
Adequate occlusion or not	15		2		17	1.0 (perfect agreement)

CTA at 6 months (range 3–9 months)	I	II	IIIa	IIIb	Total	
Modified Raymond Roy CTA	1	0	0	0	1	N/A
	I	II	III			
Raymond Roy CTA	1	0	0		1	N/A
	Yes		No			
Adequate occlusion or not	1		0		1	N/A

All imaging at 6 months (range 3–9 months)	I	II	IIIa	IIIb	Total	
Modified Raymond Roy	16	2	2	0	20	N/A
	I	II	III			
Raymond Roy	16	2	2		20	N/A
	Yes		No			
Adequate occlusion or not	18		2		20	N/A

* Two readings, one1 core laboratory and other by a local reader, were compared. For all discrepant readings, the core laboratory assessment was used. The data shows that estimates of occlusion rates were accurate when performed locally for MRA and DSA.

† Inter-reader reliability used weighted (or unweighted for 2×2) Cohen’s kKappa. N/A: nNot applicable for ‘All iImaging’ as data contains some DSA and MRA cases for same patient; insufficient data for CTA.

CTAcomputed tomography angiographyDSAdigital subtraction angiographyMRAmagnetic resonance angiographyN/Anot applicableOKMO’Kelly Marotta grading

##### Sac branch remodeling and in-stent stenosis

Of the 26/131 aneurysms (19.8%) that had arterial branches originating from the aneurysm sac, no branch was reduced in caliber at follow up and adequate occlusion was seen in 88.5% (23/26). In-stent stenosis rates are confounded but included for completeness (online supplemental table S7).

##### Aneurysm size increase

Longitudinal cross-sectional imaging was available to determine interval changes in axial diameter in 100/111 (90.1%) and 17/20 (85.0%) unruptured and ruptured aneurysms at short-term follow up, respectively. An increase in size was seen in 5/100 (5.0%) and 1/17 (5.9%) aneurysms, respectively (online supplemental table S8). At medium-term follow up, 5/13 (38.5%) unruptured aneurysms had longitudinal cross-sectional imaging and no increase in size was seen.

##### Retreatment

Two aneurysms (2/131, 1.5%) underwent further flow diversion for a remnant and an enlargement, respectively (detailed in table 3 and online supplemental table S8).

##### Subgroup analyses

A subsequent publication will present subgroup analyses including center heterogeneity; however, some initial observations were included (online supplemental table S9) after we examined the relationship between occlusion rates and (1) sidewall/bifurcation aneurysm location, (2) aneurysm neck size, and (3) aneurysm size. We found that adequate occlusion was associated with narrow-neck aneurysms at short-term follow up in the ruptured cohort.

## Discussion

### Principal findings

The adequate occlusion rate for the unruptured cohort at short-term and medium-term follow up, and also for the ruptured cohort at short-term follow up, was >90% regardless of whether analysis was on an ITT or per-protocol basis.

The major adverse event rate inevitably differed according to our analysis method: when mortality attributed to a palliative case in the unruptured cohort, or to SAH in the ruptured cohort, is excluded then the overall major adverse event rate in respective cohorts was 7.5% and 23.5%, with a 0% mortality rate for both. When all event causes are included on an ITT basis, the major adverse event rates in respective cohorts were 8.3% and 40.9%, with a 0.9% and 22.7% mortality rate.

### Comparison with studies worldwide

There were technical difficulties in 24.6% of treatments using first-version PEDVs of which 19.3% were due to stent ‘hang up’ on the pusher wire during deployment.^7^ In our study of second-version PEDVs, technical difficulties were encountered in 8.3% and 4.5% of the unruptured and ruptured cohorts respectively, plausibly demonstrating deployment improvements of the redesigned PEDV. In contrast to the 2.8% of second-version PEDVs which failed to deploy in the unruptured aneurysm cohort (0% in the ruptured cohort), all first-version PEDVs were deployed eventually in the earlier study, although not all were optimally placed due to deployment difficulty.^7^ This second-version PEDV deployment failure rate appeared to be similar to the previous generation of PEDs with Shield Technology (2.0–2.2%).^5 6^

#### Unruptured cohort

For our primary objective (treatment efficacy in unruptured aneurysms), the primary efficacy endpoint (adequate occlusion at short-term follow up) appeared higher than the previous generation of PEDs with Shield Technology when pragmatic and ITT methodology was employed using similar follow-up timepoints.^6^ Here, the adequate occlusion rates for the two studies were 90.4% and 78.8% at short-term and 92.3% and 90.3% at medium-term, respectively. The complete occlusion rates for the two studies were 71.3% and 69.2% at short-term and 76.9% and 82.7% at medium-term, respectively. When also compared with a large, single-center registry of 1000 aneurysms treated by PED, the PEDESTRIAN study, our medium-term adequate occlusion rates appeared higher (92.3% and 80.2%) and complete occlusion rates appeared comparable (76.9% and 75.8%).^16^ The short-term efficacy of first-version PEDVs, where 5% ruptured aneurysms were included, was 95.5% and 77.9% for adequate and complete occlusions, respectively.^7^

For our primary objective (treatment safety in unruptured aneurysms), the primary safety endpoint (cumulative occurrence of major adverse events at follow up) appeared similar to that seen in a pragmatic study of earlier-generation PEDs using a similar short-term follow-up timepoint and a similar adverse event classification system.^3^ Here, the major adverse event rates for the two studies were 8.3% and 6.8%, respectively. For comparison, major adverse event rates using the same classification but longer follow up in previous generation PEDs ranged from 7.4% to 13.3%.^6 10 12^ The short-term safety of first-version PEDVs, which appeared to use a different classification system, appeared to have a major adverse event rate of 4.8% on an ITT basis.^7^ The first and second PEDV version studies showed a 1.7% and 0.9% mortality rate, respectively. Both study rates were 0% on a per-protocol basis, which compares with 1.6–3.3% in the previous-generation PED studies described earlier.^3 6 10 12^

#### Ruptured cohort

For our secondary objective (treatment efficacy in ruptured aneurysms), the primary efficacy endpoint (adequate occlusion at short-term follow up) appeared higher than that seen in a study of previous-generation PEDs followed up at a similar timepoint and when applying pragmatic and ITT methodology.^17^ Here, the adequate occlusion rates for the two studies were 90.0% and 80.8% at short term, respectively. The complete occlusion rates for the two studies were 80.0% and 69.2%, respectively. When a range of follow up and study methodology was combined in a pooled analysis of 12 studies comprising 145 patients, the complete occlusion rate was similar to our study at 87.5%.^18^

The primary safety endpoint (cumulative occurrence of major adverse events at follow up) is difficult to compare directly with previous PED studies due to different and/or unclear morbidity classifications. The estimated major adverse event rate was 26.9% (7/26)^17^ and 16.5% (23/145)^18^ in the aforementioned similar study and pooled analysis, respectively. In contrast, the major adverse event rate in our study was 40.9% and 23.5% on an ITT and per-protocol basis, respectively. One adverse event was a re-rupture (1/20, 5%) in our study which compares to 2.1%^17^ and 3.8%^18^ in the respective publications mentioned previously. The mortality rate was 11.5%^17^ and 7.9%^18^ in the respective publications, and 10.5% in the IntrePED ruptured subgroup.^10^ In comparison, the mortality rate in our study was 22.7% and 0% on an ITT (including death from SAH) and per-protocol basis, respectively.

### Strengths and limitations

To the best of our knowledge, this was the first pragmatic, non-industry-sponsored study of the second PEDV version. We gave a range of outcomes on a per-protocol and ITT basis that will allow comparison with a wide range of studies.

Nonetheless, the study has several limitations. Our additional analyses demonstrated that MRA was suitable for aneurysm occlusion but not in-stent stenosis assessment. However, the number of cases used for these assessments were small. Second, while the prospective follow-up data are considered reliable, the retrospective treatment data are considered less reliable. Third, while outcomes were captured comprehensively in the short term for both adverse event and imaging follow up, there were limited outcome data for longer-term follow up. It is noteworthy, however, that there is a known rapid reduction in the likelihood of adverse events following previous-generation PED procedures,^3 6 19 20^ and in the current study no adverse events were seen ≥6 months. Furthermore, previous generations of PEDs demonstrate increased adequate and complete occlusion rates over time,^6 19 20^ a finding also noted in the current study. Fourth, while similar in size to other relevant studies of ruptured aneurysms,^18^ it is unclear how data relating to this secondary objective compare to other studies given methodological heterogeneity and a dataset enriched with small (60%) and blister (15%) aneurysms. Fifth, one drawback of a pragmatic study design is the lack of a standardized follow-up imaging protocol. While we showed that MRA appeared suitable for aneurysm occlusion assessment and therefore equivalent to DSA, there was a range of timings within our short-term and mid-term definitions limiting direct comparison with any studies with a standardized follow-up imaging protocol using ranges which are narrower.

## Conclusions

There are no published outcome studies for the second PEDV version which has superseded the limited-release first version. The second version appears easier to deploy compared with the first version. For unruptured aneurysm treatment, the second version appears to have a superior efficacy and similar safety profile to previous-generation PEDs. Overall, these are acceptable outcomes in this pragmatic and non-industry-sponsored study. Analysis of ruptured aneurysm outcomes is limited by cohort size. While efficacy appears similar to previous studies and may be acceptable, there appears to be a high rate of adverse events. To justify the routine use of PEDVs for ruptured aneurysms, evidence from further prospective outcome studies is needed.

## supplementary material

10.1136/jnis-2023-020754 online supplemental file 1

## Data Availability

Data are available upon reasonable request but UK HRA/REC compliance is needed.
