# Supplementary material for: Outcome study of the Pipeline Vantage Embolization Device (second version) in unruptured (and ruptured) aneurysms (PEDVU(R) study)
Source: J Neurointerv Surg. 2023 Dec 9;16(11):e020754. doi: 10.1136/jnis-2023-020754 (PMC11503131; doi:10.1136/jnis-2023-020754)
Supplement: online supplemental file 1 [file jnis-16-11-s001.pdf]

SUPPLEMENTAL MATERIAL

Supplementary Tables

Supplementary Table S1: Antiplatelet and anticoagulation regimens

Pre-Procedure (antiplatelets)

- **Elective Cases:**
    - Aspirin 75 mg + clopidogrel 75 mg PO OD for 7 days: 3 (37.5%)
    - Aspirin 75 mg + prasugrel 5-10 mg PO OD for 5-7 days: 2 (25%)
    - Aspirin 75-500 mg + prasugrel 20-30 mg ≥2 hours prior to procedure: 2 (25%)
    - Loading dose aspirin 150 mg + clopidogrel 300 mg 1 week before procedure then 75 mg PO OD for a week before procedure: 1 (12.5%)
- **Acute Cases:**
    - Loading dose of IV tirofiban bolus and continuous infusion, IV aspirin 500 mg + clopidogrel 600 mg PO OD: 1 (12.5%)
    - Aspirin 500 mg + prasugrel 20-30 mg ≥2 hours prior to procedure: 1 (12.5%)
    - Loading dose prasugrel 30 mg 2 hours before procedure + aspirin 500 mg IV at start of procedure: 1 (12.5%)
    - Loading dose aspirin 500 mg IV at start of procedure: 1 (12.5%)
    - Loading dose aspirin 500 mg IV and Ticagrelor 180 mg at start of procedure: 1 (12.5%)

Procedure (anticoagulation)

- Bolus of heparin (median 5000 IU IV per center), with further titration according to the activated clotting time.

Post Procedure (antiplatelets)

- **Elective Cases:**
    - Aspirin 75 mg PO OD for life + clopidogrel 75 mg PO OD for 5 to 6 months: 5 (62.5%)
    - Aspirin 75 mg PO OD for life + prasugrel 5 mg PO OD for 3 to 6 months: 4 (50%)
    - Aspirin 75 mg PO OD for 10 years + clopidogrel 75 mg PO OD for 9 months: 1 (12.5%)
- **Acute Cases (if different than elective post procedural regimen):**
    - Aspirin 75 mg PO OD for 1 year, occasionally for life: 1 (12.5%)
    - Aspirin 75mg OD for 1 year + Ticagrelor 90 mg BD for 3 months: 1 (12.5%)

Data shown is number of centers (relative frequency in %)
Relative frequency related to the number of centers (n = 8)

Supplementary Table S2: Interventional routes and catheters

**Approach(es)**	Transfemoral: 8 (100%)<br>Radial: 7 (87.5%)<br>Ulnar: 1 (12.5%)
**Large catheter(s)**	Neuron MAX 0.088 (Penumbra, Alameda, USA): 8 (100%)<br>Benchmark 6 Fr (Penumbra): 4 (62.5%)<br>Rist 0.079 (Medtronic Neurovascular, Minneapolis, USA): 2 (25%)<br>Fubuki 8 Fr (Asahi, Tokyo, Japan): 1 (12.5%)<br>Inifinity 8 Fr (Stryker, Kalamazoo, USA): 1 (12.5%)
**Intermediate catheter(s) (if used)**	Navien 0.058 (Medtronic Neurovascular): 8 (100%)<br>Navien 0.072 (Medtronic Neurovascular): 4 (50%)<br>Sofia 5 Fr or 6 Fr (MicroVention, Aliso Viejo, USA): 1 (25%)<br>Catalyst 5 Fr (Stryker): 2 (25%)
**Deploying microcatheter(s)**	Phenom 0.027 (Medtronic Neurovascular): 8 (100%)<br>Phenom 0.021 (Medtronic Neurovascular): 4 (50%)

Data shown is number of centers (relative frequency in %)
Relative frequency related to the number of centers (n = 8)

Supplementary Table S3: O'Kelly Marotta (OKM) grading immediately after PEDV deployment (per-protocol analysis)

Unruptured Cohort

OKM grading	A1	A2	A3	B1	B2	B3	C1	C2	C3	D
**Number of cases**	25	10	22	5	7	21	6	4	6	5
**Relative frequency (%)**	22.5	9.0	19.8	4.5	6.3	18.9	5.4	3.6	5.4	4.5

Ruptured Cohort

OKM grading	A1	A2	A3	B1	B2	B3	C1	C2	C3	D
**Number of cases**	9	5	2	0	0	3	0	0	0	1
**Relative frequency (%)**	45.0	25.0	10.0	0.0	0.0	15.0	0.0	0.0	0.0	5.0

Relative frequency related to the number of aneurysms (n = 111 for unruptured and n = 20 for ruptured).
Core laboratory assessment.

Supplementary Table S4: Details of clinical follow up of survivors following procedure. Survivors include those recorded on a per protocol basis and additionally include those 3 patients requiring alternative treatment for failed PEDV deployment in the unruptured cohort. Numbers reflect date of procedure and time followed up so far, as opposed to loss to follow up.

Follow up	Unruptured Cohort (n/n, %)	Ruptured Cohort (n/n, %)
**Peri-procedural 30 days**	107/107, 100.0	17/17, 100.0
**≥ 3 (post-procedural)**	107/107, 100.0	17/17, 100.0
**≥ 6 (post-procedural)**	75/107, 70.1	13/17, 76.5
**≥ 9 (post-procedural)**	20/107, 18.7	4/17, 23.5
**≥ 12 (post-procedural)**	8/107, 7.5	1/17, 5.9

Supplementary Table S5: Association between neurological adverse events (on an intention-to-treat basis) and anti-platelets protocol

	Unruptured Cohort (n = 108)	Major / Minor	Antiplatelet Regimen		VerifyNow
			Pre-Procedural	Post Procedural	
**Peri-procedural neurological adverse events**	Death*: 1 (0.9%)	**Major**	Aspirin 75 mg + prasugrel 5 mg PO OD for 7 days	Aspirin 75 mg PO OD for life + prasugrel 5 mg PO OD for 3 to 6 months	No
	Ischemic stroke: 7 (6.5%)				
	- Case 1	Minor	Aspirin 75 mg + clopidogrel 75 mg PO OD for 7 days	Aspirin for life	No
	- Case 2	**Major**	Aspirin 75 mg + clopidogrel 75 mg PO OD for 7 days	Aspirin 75 mg PO OD for life + clopidogrel 75 mg PO OD for 5 months	**Yes**
	- Case 3	Minor	Loading dose aspirin 150 mg + clopidogrel 300 mg 1 week before procedure then 75 mg PO OD for a week before procedure	Aspirin 75 mg PO OD for life + clopidogrel 75 mg PO OD for 5 months	No
	- Case 4	Minor	Aspirin 75 mg + prasugrel 5 mg PO OD for 7 days	Aspirin 75 mg PO OD for life + prasugrel 5 mg PO OD for 3 to 6 months	No
	- Case 5	Minor	Aspirin 500 mg + prasugrel 20-30 mg ≥2 hours prior to procedure	Aspirin 75 mg PO OD for life + prasugrel 5 mg PO OD for 6 months	**Yes**
	- Case 6	Minor	Aspirin 500 mg + prasugrel 20-30 mg ≥2 hours prior to procedure	Aspirin 75 mg PO OD for life + prasugrel 5 mg PO OD for 6 months	**Yes**

	- Case 7	**Major**	Aspirin 75 mg PO OD for life + prasugrel 5 mg PO OD for 3 to 6	Aspirin 75 mg PO OD for life + prasugrel 5 mg PO OD for 3 to 6 months	**Yes**
	Cranial neuropathy: 2 (1.9%)				
	- Case 1	**Major**	Aspirin 75 mg + clopidogrel 75 mg PO OD for 7 days	Aspirin 75 mg PO OD for life + clopidogrel 75 mg PO OD for 6 months	No
	- Case 2	**Major**	Aspirin 75 mg PO OD for life + prasugrel 5 mg PO OD for 3 to 6	Aspirin 75 mg PO OD for life + prasugrel 5 mg PO OD for 3 to 6 months	**Yes**
	Intraparenchymal hemorrhage: 1 (0.9%)	**Major**	Aspirin 75 mg + clopidogrel 75 mg PO OD for 7 days	Aspirin 75 mg PO OD for life + clopidogrel 75 mg PO OD for 6 months	No
**Post-procedural neurological adverse events**	Ischemic stroke: 3 (2.8%)				
	- Case 1	Minor	Aspirin 75 mg + clopidogrel 75 mg PO OD for 7 days	Aspirin 75 mg PO OD for 10 years + clopidogrel 75 mg PO OD for 9 months	No
	- Case 2	Minor	Aspirin 75 mg + clopidogrel 75 mg PO OD for 7 days	Aspirin 75 mg PO OD for life + clopidogrel 75 mg PO OD for 6 months	No
	- Case 3	Minor	Aspirin 75 mg + clopidogrel 75 mg PO OD for 7 days	Aspirin 75 mg PO OD for life + clopidogrel 75 mg PO OD for 5 months	**Yes**
	Cranial neuropathy: 1 (0.9%)	**Major**	Aspirin 75 mg + clopidogrel 75 mg PO OD for 7 days	Aspirin 75 mg PO OD for life + clopidogrel 75 mg PO OD for 6 months	No
	Parent artery occlusion†: 1 (0.9%)	**Major**	Aspirin 75 mg + clopidogrel 75 mg PO OD for 7 days	Aspirin 75 mg PO OD for life + clopidogrel 75 mg PO OD for 5 months	**Yes**
	Aneurysm growth with mass effect: 1 (0.9%)	**Major**	IV bolus of tirobifan followed by continuous infusion.	Loaded with 500 mg aspirin and 600 mg of clopidogrel.	No

Aspirin for life, clop for 6 months

	Ruptured Cohort (n = 22)	Major / Minor	Antiplatelet Regimen		VerifyNow
			**Pre-Procedural**	**Post Procedural**	
**Peri-procedural neurological adverse events**	Death‡: 5 (22.7%)<br>- Case 1	**Major**	Loading dose of 500 mg IV Aspirin	N/A	No
	- Case 2	**Major**	Loading dose of 500 mg IV Aspirin	N/A	No
	- Case 3	**Major**	Loading dose of 500 mg IV Aspirin	N/A	No
	- Case 4	**Major**	Loading dose of 500 mg IV Aspirin	N/A	No
	- Case 5	**Major**	Loading dose prasugrel 30 mg 2 hours before procedure + aspirin 500 mg IV at start of procedure	Aspirin 75 mg PO OD for life + clopidogrel 75 mg PO OD for 5 months	No
	Ischemic stroke‡: 4 (18.2%)<br>- Case 1	**Major**	Loading dose prasugrel 30 mg 2 hours before procedure	Aspirin 75 mg PO OD for 1 year	**Yes**
	- Case 2	Minor	Loading dose prasugrel 30 mg 2 hours before procedure	Aspirin 75 mg PO OD for 1 year	**Yes**
	- Case 3	**Major**	Loading dose prasugrel 30 mg 2 hours before procedure + aspirin 500 mg IV at start of procedure	Aspirin 75 mg PO OD for life + clopidogrel 75 mg PO OD for 5 months	No
	- Case 4	**Major**	Loading dose aspirin 500 mg IV and ticagrelor 180 mg at start of procedure	Aspirin 75mg OD for 1 year + ticagrelor 90 mg BD for 3 months	No
	Second subarachnoid hemorrhage§: 1 (4.5%)	**Major**	IV bolus of tirobifan followed by continuous infusion.	Aspirin 75 mg PO OD for life + clopidogrel 75 mg PO OD for 6 months	No

Peri-procedural: occurring < 30 days after embolization.

Post-procedural: occurring ≥ 30 days.

"Major" adverse event: a persistent clinical deficit at 7 days following the event. "Minor" adverse event: events that resolved within 7 days with no clinical sequelae.

* Adjudication concluded death inevitable without PEDV implantation

† No neurological clinical features but included as neurological adverse event according to IntrePED methodology[10].

‡ Adjudication concluded death more likely to be attributable to presenting subarachnoid hemorrhage than PEDV implantation; data was less clear for ischemic stroke.

§ Patient had ruptured ICA para-ophthalmic segment of ICA aneurysm. Pre-operative IV tirofiban with infusion. Underwent PEDV deployment, adjunctive coiling and balloon angioplasty. Loaded with aspirin and clopidogrel. Re-rupture with immediate hydrocephalus in recovery. Given 2 pools of platelets and underwent extra ventricular drain insertion. Subsequently underwent ventriculo-peritoneal shunt insertion. Complete occlusion of aneurysm at follow up.

Supplementary Table S6: Details of imaging hardware, routine imaging follow-up protocols and imaging acquisition parameters. Computed tomographic angiography was not routinely used.

**Fluoroscopy model**	Allura Xper FD & Azurion (Philips Healthcare, Amsterdam, Netherlands): 5 (62.5%)<br>Artis & Icono (Siemens, Munich, Germany): 4 (50%)
**DSA follow-up frequency**	6 months: 3 (37.5%)<br>24 months: 1 (12.5%)
**MRI model**	MAGNETOM Aera 1.5 T, MAGNETOM Sola 1.5 T, MAGNETOM Skyra 3 T, MAGNETOM Avanto 1.5 T & MAGNETOM Vida 3 T (Siemens): 6 (75%)<br>Ingenia 1.5 T (Philips Healthcare): 2 (25%)<br>SIGNA 1.5 T HDx, SIGNA Premier 3 T & SIGNA Artist 1.5 T (General Electric, Boston, Massachusetts, USA): 2 (25%)
**MRA follow-up frequency**	6 months: 7 (87.5%)<br>18 months: 5 (62.5%)<br>24 months: 6 (75%)<br>36 months: 1 (12.5%)<br>48 months: 1 (12.5%)<br>60 months: 3 (37.5%)
**MRA Exam Technique**	**Range of Values**
**Technique used**	3D-TOF-MRA: 7 (87.5%)<br>CEMRA: 1 (12.5%)
**TR**	18 to 39 ms
**TE**	2.5 to 7 ms
**Flip angle**	18° to 25°
**Matrix**	228 x 228 to 512 x 512
**FOV**	18 to 21 cm
**Slice thickness**	0.5 to 1.4 mm

CEMRA: Contrast Enhanced Magnetic Resonance Angiography, DSA: Digital Subtraction Angiography, MRA: Magnetic Resonance Angiography, TE: Time to Echo, TOF: Time-of-Flight, TR: Repetition Time.
DSA machine vendors and MRA machine details are displayed in number of centers (relative frequency in %).
Relative frequency related to the number of centers (n = 8).

Supplementary Table S7: Data on in-stent stenosis is included for completeness, but is confounded. The DSA subgroup is enriched as it includes cases of suspected in-stent stenosis on MRA. MRA insensitive to in-stent stenosis diagnosis due to susceptibility.

Follow up*	in-stent stenosis < 50% (n/n, %)	in-stent stenosis ≥50% (n/n, %)
**Short-term DSA**	3/14, 21.4	1/14, 7.1
**Medium-term DSA**	2/8, 25.0	0/8, 0
**Short-term MRA**	2/94, 2.1	2/94, 2.1
**Medium-term MRA**	0/5, 0	0/5, 0

* All cases unruptured except for 1 short-term DSA < 50%.
DSA: Digital Subtraction Angiography, MRA: Magnetic Resonance Angiography

Supplementary Table S8: Longitudinal cross-sectional imaging at short-term follow up showing interval change in axial diameter.

Interval increase (mm)*	Rupture status	Index case a retreatment?	Adjuncts used	MRRC	Symptomatic	Treatment
2.5	Ruptured	No	Coils	IIIa	No	No
2.5	Unruptured	No	Coils	I	No	No
2.5	Unruptured	Coiled	No	IIIa	No	No
3.5	Unruptured	Stent-coiled	No	IIIa	No	No
4	Unruptured	No	Coils	I	No	No
10	Unruptured	Coiled	Coils	II	Yes†	Yes†

MRRC = Modified Raymond Roy classification scale

* Interval increase > 2 mm to nearest 0.5 mm

† Described above as a post-procedural neurological adverse event (temporal hemianopia resolved with 14 days of steroids) and also listed as a re-treatment. Enlargement was seen on short-term imaging follow up at 6 months, therefore the patient underwent endovascular treatment with a second PEDV)

Supplementary Table S9 Subgroup analysis (Fisher exact tests) performed to examine the relation between occlusion rates and (1) sidewall/bifurcation aneurysm location, (2) aneurysm neck size, and (3) aneurysm size.

	**Sidewall vs Bifurcation**	**Neck size**	**Aneurysm size***	
	P value	*P value*	*P value*	
			P_A	P_B
**Unruptured**				
Short-term occlusion				
Adequate	1.00	0.75	0.56	0.56
Complete	0.19	0.06	0.06	0.06
Medium-term occlusion				
Adequate	1.00	0.46	1.00	0.62
Complete	1.00	0.56	1.00	1.00
**Ruptured**				
Short-term occlusion				
Adequate	1.00	**0.03**	0.07	0.07
Complete	1.00	0.16	0.06	0.06

* Freeman-Halton extension of the Fisher exact probability test for a two-rows by four-columns contingency table, providing that the total size of the data set is no greater than n = 120. The test will yield two probability values, PA and PB, defined as follows:
- PA = the probability of the observed array of cell frequencies plus the sum of the probabilities of all other cell-frequency arrays (such as would be consistent with the observed marginal totals) that are equal to or smaller than the probability of the observed array.
- PB = the probability of the observed array of cell frequencies plus the sum of the probabilities of all other cell-frequency arrays (such as would be consistent with the observed marginal totals) that are smaller than the probability of the observed array.
- PA and PB are both non-directional (two-tailed) probabilities.

Supplementary Figures

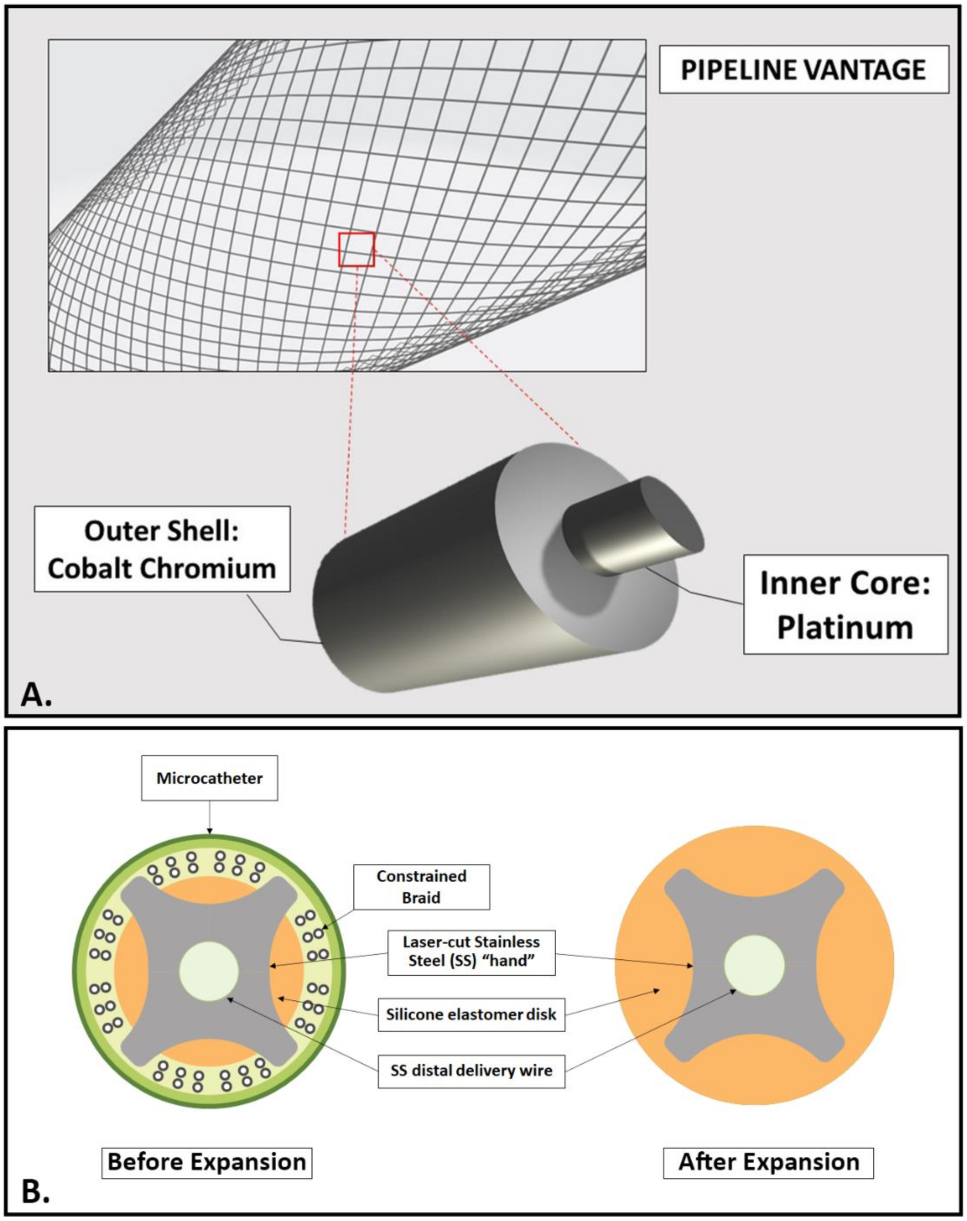

Supplementary Figure S1: First version PEDVs were introduced in 2020 and limited to a few centers globally to allow feedback. The key change being that separate monofilament platinum and cobalt chromium (CoCr) wires of the FLEX version were replaced by 48 thinner drawn filled tube (DFT) wires. For the larger diameter PEDVs, 16 CoCr wires were additionally incorporated (total of 64 wire braids). With the aim that distal release would be fast, the polytetrafluoroethylene (PTFE) sleeves in PEDV were made thinner and positioned differently. The proximal detachment zone was also redesigned to improve delivery. Another change was to replace a hollow hypotube with a continuous corewire. The changes enabled 0.021 microcatheters to be used with smaller PEDVs as opposed to all PEDVs being deployed with 0.027 inch microcatheters. Version 1 was modified because of technical procedural complications consisting mainly of 'hanging-up' of the device on the pusher wire preventing proper PEDV release. Specifically, the modifications consisted of increasing the CoCr to platinum ratio in the DFT wires for certain diameters, and adding small silicon plates adjacent to the gearwheel-like plate at the proximal portion of the interface between the PEDV and the pusher wire preventing device 'hang-up'. (**A**) Pipeline vantage (Fourth generation – version 2) new design was released globally and featured a new integrated wire design which involves the new DFTs with a cobalt chromium (CoCr) shell and an inner platinum core. Whilst DFTs were introduced for version 1, the CoCr to platinum ratio was modified for certain sizes. (**B**) Version 2 also introduces silicone elastomer disk which upon expansion prevents the re-sheathing mechanism from engaging with the pores of the deployed braid.

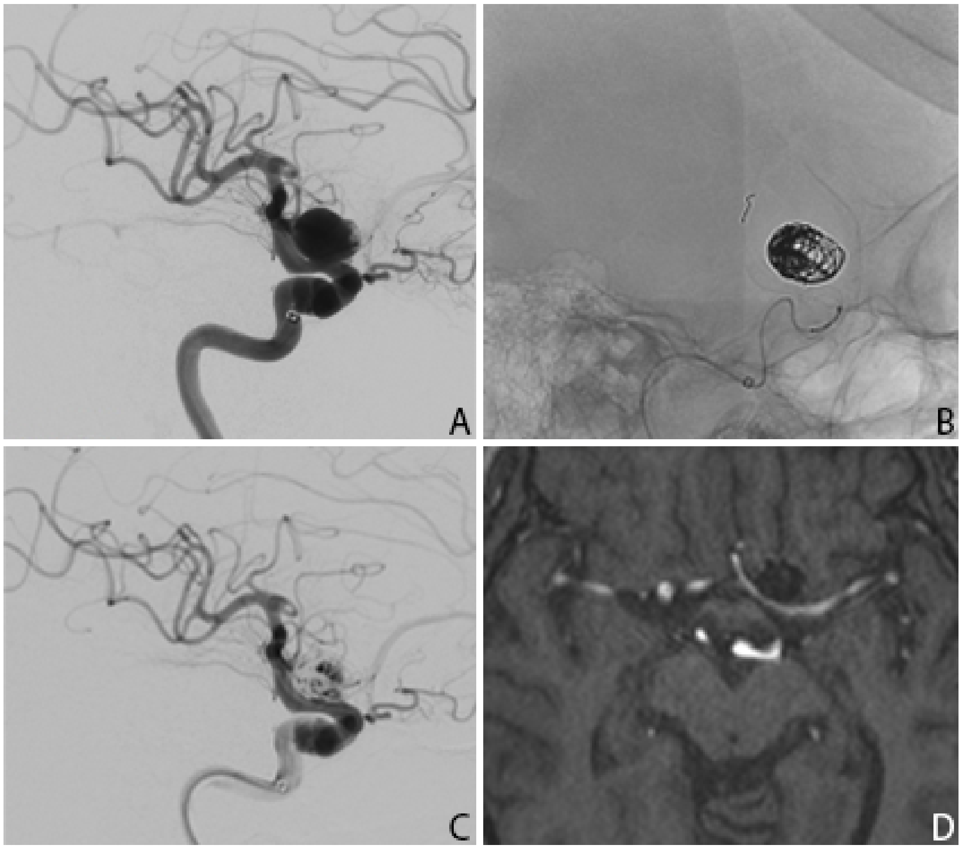

Supplementary Figure S2: Illustrative example of PEDV deployment and follow-up imaging. (**A**) Digital subtraction angiography (DSA) showing an aneurysm at the paraophthalmic segment of the left ICA. (**B**) Procedural single-shot radiograph showing deployment of the PEDV shortly after the aneurysm had been coiled. (**C**) Immediate post-embolization DSA showing O'Kelly Marotta scale of B1. (**D**) Magnetic Resonance Angiography with Time-of-Flight (MRA TOF) performed 6 months later showing modified Raymond Roy scale of I indicating complete occlusion of the aneurysm.

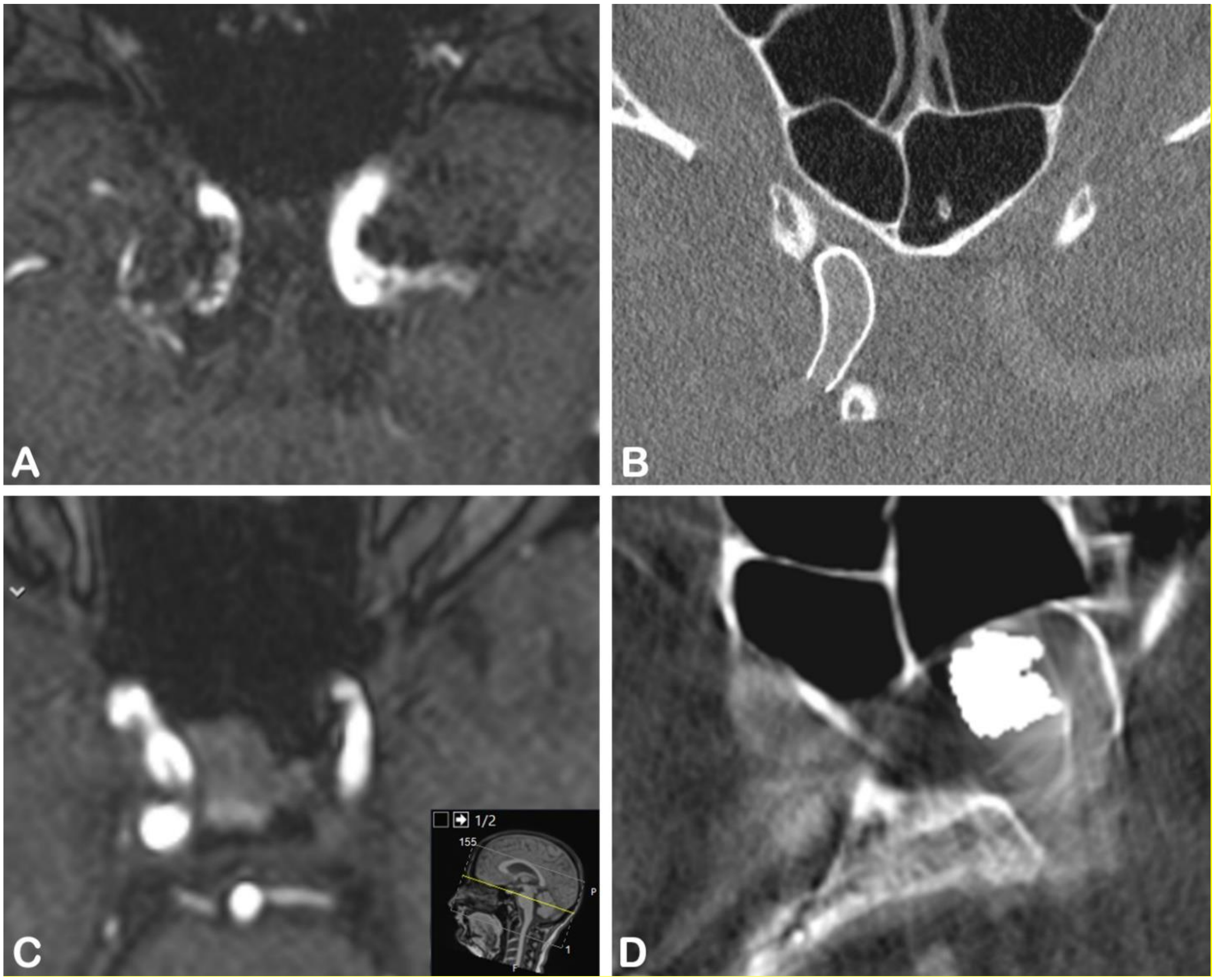

Supplementary Figure S3: Illustrative examples of susceptibility artifact on follow up imaging of the PEDV which appear more marked compared to previous generations of PEDs and are plausibly caused by the change in design. Two cases are demonstrated; Case 1: (Figures A&B) Magnetic Resonance Angiography with Time-of-Flight (MRA TOF) showing intra-luminal susceptibility artifact of a PEDV placed in right ICA (Fig. A), with suspected in-stent stenosis. (Fig. B) Follow up IV Digital Subtraction Angiography (DSA) employing 3D flat panel tomography showing normal luminal opacification within the PEDV with no in-stent stenosis. Case 2: (Figs. C & D) shows similar changes in a PEDV placed in the left ICA. MRA TOF (Fig. C) shows susceptibility artifact of the PEDV with suspected in-stent stenosis which showed normal luminal opacification within the PEDV with no in-stent stenosis on follow up IV DSA (Fig. D). In case 2 the aneurysm has also been coiled as demonstrated in Figure D.
